# The effects of continued azacitidine treatment cycles on response in higher risk patients with myelodysplastic syndromes: an update

**DOI:** 10.3332/ecancer.2008.118

**Published:** 2008-12-08

**Authors:** LR Silverman, P Fenaux, GJ Mufti, V Santini, E Hellström-Lindberg, N Gattermann, G Sanz, AF List, SD Gore, JF Seymour, J Backstrom, D McKenzie, CL Beach

**Affiliations:** 1 Division of Hematology, Mount Sinai School of Medicine, New York, NY, USA; 2 Hôpital Avicenne, Université Paris 13, Bobigny, France; 3 Department of Haematological Medicine, Kings College London, London, UK; 4 Hematology, Azienda Ospedaliera Careggi, Firenze, Italy; 5 Karolinska University Hospital, Stockholm, Sweden; 6 Heinrich-Heine University, Düsseldorf, Germany; 7 Department of Hematology, Hospital Universitario La Fe, Valencia, Spain; 8 H. Lee Moffitt Cancer Center and Research Institute, Tampa, FL, USA; 9 The Sidney Kimmel Comprehensive Cancer Center, Johns Hopkins, Baltimore, MD, USA; 10 Department of Haematology, Peter MacCallum Cancer Institute, Victoria, Australia; 11 Celgene, Overland Park, KS, USA

## Abstract

The international, phase III, multi-centre AZA-001 trial demonstrated azacitidine (AZA) is the first treatment to significantly extend overall survival (OS) in higher risk myelodysplastic syndromes (MDS) patients (Fenaux (2007) *Blood* **110** 817). The current treatment paradigm, which is based on a relationship between complete remission (CR) and survival, is increasingly being questioned (Cheson (2006) *Blood* **108** 419). Results of AZA-001 show CR is sufficient but not necessary to prolong OS (List (2008) *Clin Oncol* **26** 7006). Indeed, the AZA CR rate in AZA-001 was modest (17%), while partial remission (PR, 12%) and haematological improvement (HI, 49%) were also predictive of prolonged survival. This analysis was conducted to assess the median number of AZA treatment cycles associated with achievement of first response, as measured by IWG 2000-defined CR, PR or HI (major + minor). The number of treatment cycles from first response to best response was also measured.

## Methods

Patients (pts) with higher risk MDS (FAB: RAEB, RAEB-T, or CMML and IPSS: Int-2 or High) were included. Pts were randomized to AZA (75 mg/m^2^/d SC × 7d q 28d) or to a conventional care regimen (CCR). AZA treatment was continued up to disease progression (or unacceptable toxicity), regardless of haematological response. Erythropoiesis stimulating agents were not allowed.

## Results

In all, 358 pts were randomized (179 to AZA and 179 to CCR). Of the 179 AZA pts, 91 (51%) achieved a CR, PR or HI. For the 91 pts who achieved an IWG response, the median number of cycles to first response was three (range: 1–22), 81% of pts achieved a first response by six cycles, and 90% achieved a first response by nine cycles. For 57% of responders (*n*=52), their first response was their best response; the remaining 43% (*n*=39) had an improvement in their response status at a median of approximately four additional treatment cycles (range 1–11 treatment cycles) after their first response.

## Conclusions

While many pts achieving a haematological response with AZA do so in early treatment cycles, continued AZA dosing can further improve pt responses. In the AZA-001 study, a significant OS benefit was observed compared with CCR. In this study, AZA pts received a median of nine treatment cycles (range 1–39). For those achieving a response of HI or better, 90% did so by nine cycles; more than 40% of responders later achieved an improved response. In the absence of unacceptable toxicity or disease progression, continued AZA treatment is appropriate and may maximize patient benefit.

